# Enhanced Reduction of Graphene Oxide on Recyclable Cu Foils to Fabricate Graphene Films with Superior Thermal Conductivity

**DOI:** 10.1038/srep14260

**Published:** 2015-09-25

**Authors:** Sheng-Yun Huang, Bo Zhao, Kai Zhang, Matthew M. F. Yuen, Jian-Bin Xu, Xian-Zhu Fu, Rong Sun, Ching-Ping Wong

**Affiliations:** 1 Shenzhen Institutes of Advanced Technology, Chinese Academy of Sciences, Shenzhen, 518055, P. R. China; 2 Shenzhen High Density Electronic Packaging and Device Assembly Key Laboratory Shenzhen, 518055, China; 3 Department of Mechanical Engineering, Hong Kong University of Science and Technology, Hong Kong, China; 4 Department of Electronics Engineering, The Chinese University of Hong Kong, Hong Kong, China; 5 School of Materials Science and Engineering, Georgia Institute of Technology, Atlanta, Georgia 30332-0245, United States

## Abstract

Large-area freestanding graphene films are facilely fabricated by reducing graphene oxide films on recyclable Cu foils in H_2_-containing atmosphere at high temperature. Cu might act as efficient catalysts for considerably improved reduction of graphene oxide according to the SEM, EDS, XRD, XPS, Raman and TGA results. Comparing to the graphene films with ~30 μm thickness reduced without Cu substrate at 900 °C, the thermal conductivity and electrical conductivity of graphene films reduced on Cu foils are enhanced about 140% to 902 Wm^−1^K^−1^ and 3.6 × 10^4^ S/m, respectively. Moreover, the graphene films demonstrate superior thermal conductivity of ~1219 Wm^−1^K^−1^ as decreasing the thickness of films to ~10 μm. The graphene films also exhibit excellent mechanical properties and flexibility.

Modern electronics including smart phone, tablet PC, laptop, and LED lamp have been currently developing very fast for wide applications. Considerable heat emissions would be generated in these multifunctional portable or high power electronic devices. Thus effective thermal management is becoming extremely crucial to remove the large heat flux and ensure high performance and long lifetime reliability for these electronic devices^1^,^2^. High thermal conductive materials mainly of thermal interface materials (TIMs) and lateral heat spreaders play important roles in the thermal management to dissipate heat efficiently.

Graphene, a two-dimensional material, has been intensively studied as a promising thermal conductive material for thermal management because of its unique advantages including the highest in-plane thermal conductivity, excellent mechanical property, low density and thermal expansion coefficient^3^,^4^,^5^,^6^,^7^,^8^,^9^,^10^,^11^,^12^,^13^. Graphene sheets could be used as thermal conductive filler for the polymer TIMs to fill the gaps between the heat sources and heat sinks, thus to increase the thermal transfer efficiency. Graphene films (papers) then could be directly used as lateral heat spreaders to remove local hot spot by dissipating the heat along the film’s plane. Comparing to the graphene prepared by chemical vapor deposition (CVD) or physical exfoliation method, it is a cost-effective and easily industrialized procedure to produce graphene in large amount by reduction of graphene oxide (GO) which derived from cheap and abundant graphite raw material. Moreover, graphene oxide could be easily fabricated as macroscopic films to obtain large area free-standing graphene films after reduction using chemical agent or just thermal annealing^14^,^15^,^16^,^17^,^18^,^19^. However, the conventional reduced graphene oxide usually showed very low thermal conductivity due to the defect from graphene oxide and hard enough reduction, otherwise it was graphitized at very high temperature such as 2000 °C to obtain high thermal conductivity of ~1100 Wm^−1^K^−1^ for 8.4 μm thick films^4^.

Herein, we report a facile approach to fabricate large-area graphene films with excellent thermal and electrical conductivity from GO film (GOF) at low temperature of 900 °C using Cu foils as recyclable catalytic substrates in the H_2_-containing atmosphere. The GOF could be considerably reduced on Cu foils than that without Cu catalytic substrates. The as-prepared ~10 μm thick flexible graphene films demonstrate super high thermal conductivity of ~1219 Wm^−1^K^−1^.

## Results

**Figure 1:**
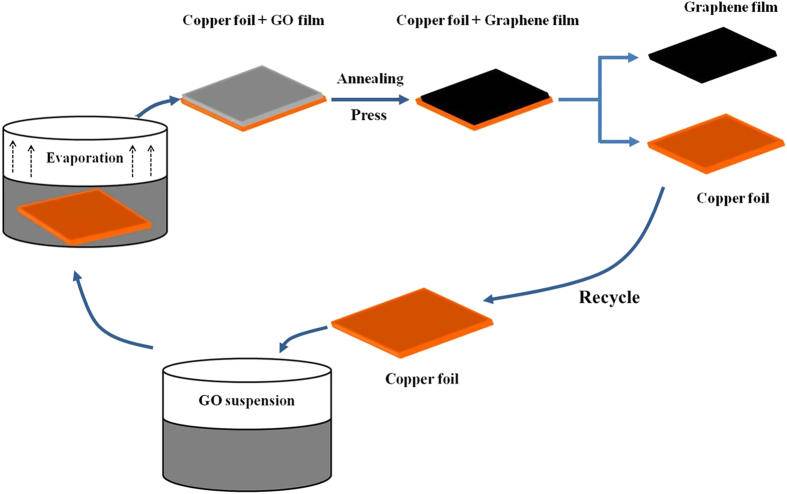
Fabrication of GEF process. Schematic drawings illustrating the procedures to the deposition GOF on Cu foil substrate and subsequent mechanical press and thermal annealing of the film.

A typical procedure for preparation of graphene films on recyclable Cu foils is illustrated in Fig. 1. First, the Cu foil was immersed into the graphene oxide (GO) hydrogel (3–5 mg/mL) obtained by using a modified Hummers method. Subsequently, the GO hydrogel was kept in evaporation of water under appropriate heating (50 °C) until drying. Afterwards, a dark brown graphene oxide film (GOF) deposited on Cu foil was obtained and hot pressed at 150 °C for 1 h. The thickness could be well controlled from several to several ten micrometers by adjusting the volume and concentration of GO suspension. More importantly, it could easily manufacture large-area GOF by this direct evaporation method. Thermal annealing at 900 °C in 5%H_2_-Ar was further employed for the reduction of the GOF. Finally, a free-standing and paper-like graphene film was obtained by easily peeling off from Cu foil, and the Cu foil could be also recycled in the next fabrication of graphene films.

**Figure 2:**
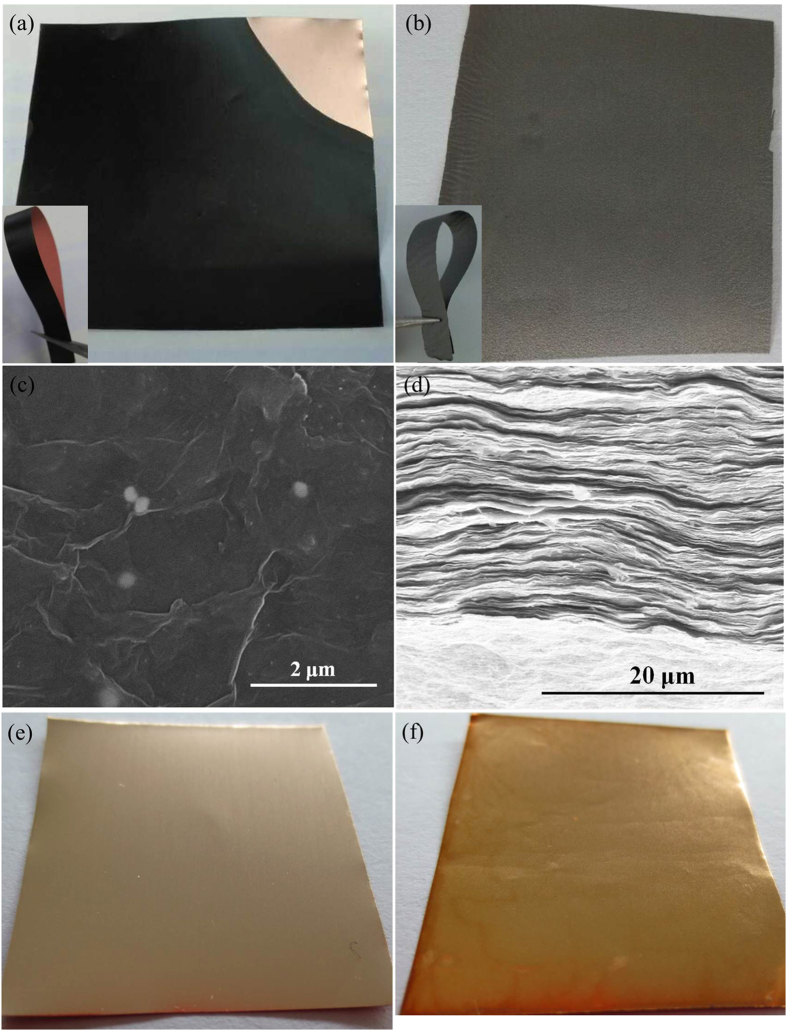
Morphology characterization of GOF, GEF-C and Cu foil. (**a**) Photograph of GOF-Cu foil; (**b**) Photograph of GEF-C; (**c**) SEM image of the surface morphology of GEF-C; (**d**) SEM image of the cross-sectional morphology of GEF-C; (**e**) Photograph of pure Cu foil before GO deposition; (**f**) Photograph of Cu foil after peeling of graphene film.

The morphology and microstructure were observed during the fabrication of graphene film with recyclable Cu foil substrate. Figure 2a represents a digital photo of a piece of 36 cm^2^ large black GOF deposited on the Cu foil substrate. The patterns and area of GOF could be easily controlled by adjusting the shape and size of Cu foil substrate. After mechanical press and thermal annealing, the film exhibited shiny graphite luster (Fig. 2b), suggesting the efficient reduction. The insets of Fig. 2a,b show that GO-Cu foil and graphene film could be easily bended by 180 ° without destruction, indicating their excellent flexibility. The in-plane and cross-sectional images of GEF-C were obtained by scanning electron microscopy (SEM) as shown in Fig. 2c,d. The surface of graphene film was relatively smooth. The fracture edge of the film revealed well aligned layer-by-layer nanostructure of graphene sheets. Figure 2e,f illustrate the optical micrographs of pure Cu foil before GO deposition and after peeling off graphene film. Compared with pure Cu foil, the Cu foil coated with GOF was only brighter after thermal annealing at 900 °C. It could be reused to fabricate graphene films again. Higher temperature such as 1000 °C was also tried to reduce the GOF on Cu foils. However, the Cu foils were melted to Cu balls (Fig. 1S) which might be resulted from the interaction of GO and Cu foils at high temperature although the melt temperature of Cu is 1083 °C. So we chose 900 °C here in order to reduce GO and prevent Cu foils efficiently.

**Figure 3:**
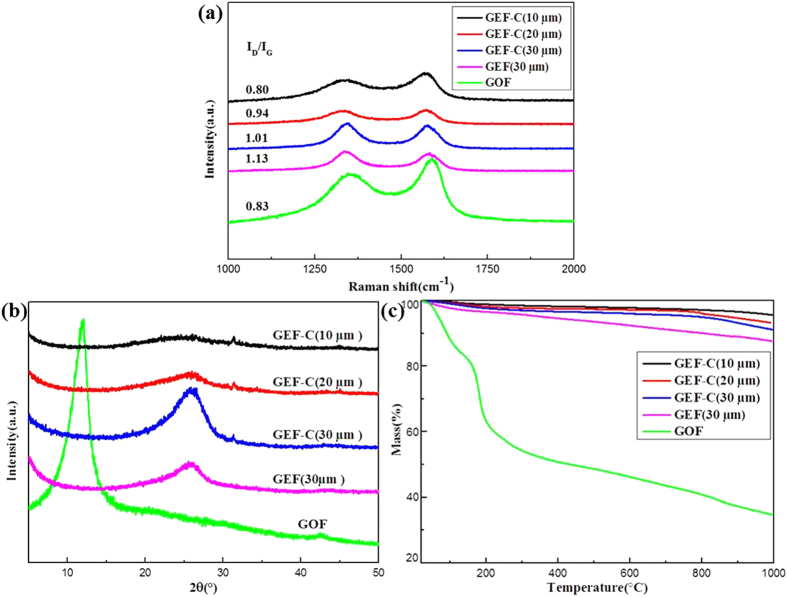
Structural characterization of GOF, GEF, and GEF-C. (**a**) Raman spectra, (**b**) XRD patterns and (**c**) TGA curves.

Raman spectroscopy was employed to reveal the effect of Cu foil on GO reduction, as shown in Fig. 3a. According to the previous work, GO sheets displayed a remarkable G peak at around 1593 cm^−1^–1606 cm^−1^ before reduction^20^,^21^,^22^. It has been reported that the decrease in G band energy indicates the development of long-range and in-plane graphene order^23^,^24^. Figure 3a shows that the G peak position shifted to lower wavenumber for GEF-C films with thinner thickness, at 1576.4 cm^−1^ for 30 μm, 1575.1 cm^−1^ for 20 μm and 1573.4 cm^−1^ for 10 μm, respectively. For the GEF film with 30μm derived from annealing at 900 °C without Cu substrate, the G peak still maintained at a relatively higher wavenumber at 1581.2 cm^−1^. The results suggest the efficient recovery of the graphene structure from GO on Cu substrate after reduction at 900 °C, especially for the thinner films. Compared with GEF, the *I*_D_/*I*_G_ ratio decreased notably from 1.13 to 1.01, 0.94 and 0.80 of GEF-C, implying that fewer defects created during thermal reduction^18^,^19^. It might be due to the Cu catalytic function for de-oxygenation in the GO reduction. Hydrogen might not only remove the oxygen-containing functional groups in GO but also activate the catalytic property of Cu at high temperature according the previous report about fabrication of graphene using hydrocarbon or solid carbon feedstock^25^,^26^. The structure evolution of sample was further investigated by X-ray diffraction (XRD). As shown in Fig. 3b, the diffraction peak of GOF spectrum at around 2θ = 11.8 ° (*d*-spacing = 0.75 nm) exhibited that GOF had a layered structure. The large interlayer distance of GOF was attributed to the formation of oxygen-containing functional groups, allowing water molecules to intercalate between the layers which increased the distance between the layers^27^. However, this peak disappeared in the XRD patterns of GEF and GEF-C, resulting from the extensive reduction of GOF during thermal annealing in H_2_-containing atmosphere. In contrast, the interlayer distance of GEF-C decreased (around 26.2 ° to 26.4 °, *d*-spacing = 0.34 nm) relative to GEF (around 25.6 °, *d*-spacing = 0.35 nm). It was assigned to larger removal of oxygen-containing functional groups and more regular packing of graphene layer for the graphene oxide reduction on the Cu foil. Besides, elemental composition of the films was further analyzed by elemental analysis (Table 1). The C/O atomic ratio was increased from 1.8 of GOF to 10.1 of GEF 30 μm and 14.8 of GEF-C 30 μm, 19.8 of GEF-C 20 μm, 23.4 of GEF-C 10 μm, respectively. The results indicate that Cu foil greatly enhanced the reduction of GO to graphene, facilitating the removal of oxygen-containing functional groups and restoration of regular graphite lattice. The thermochemistry of GOF, GEF, and GEF-C were further characterized by thermogravimetric spectrometry in nitrogen atmosphere (Fig. 3c). As listed in Table 1, the residual carbon ratios increased progressively from 34.8% for GOF to 86.4% for GEF and 91.5%, 93.2%, 95.2% for GEF-C with thickness of 30 μm, 20 μm and 10 μm, respectively. The results also suggest the much effective reduction of GO to graphene film on the Cu foil substrate at high temperature in H_2_-containing atmosphere, especially for the thinner GO films.

**Figure 4:**
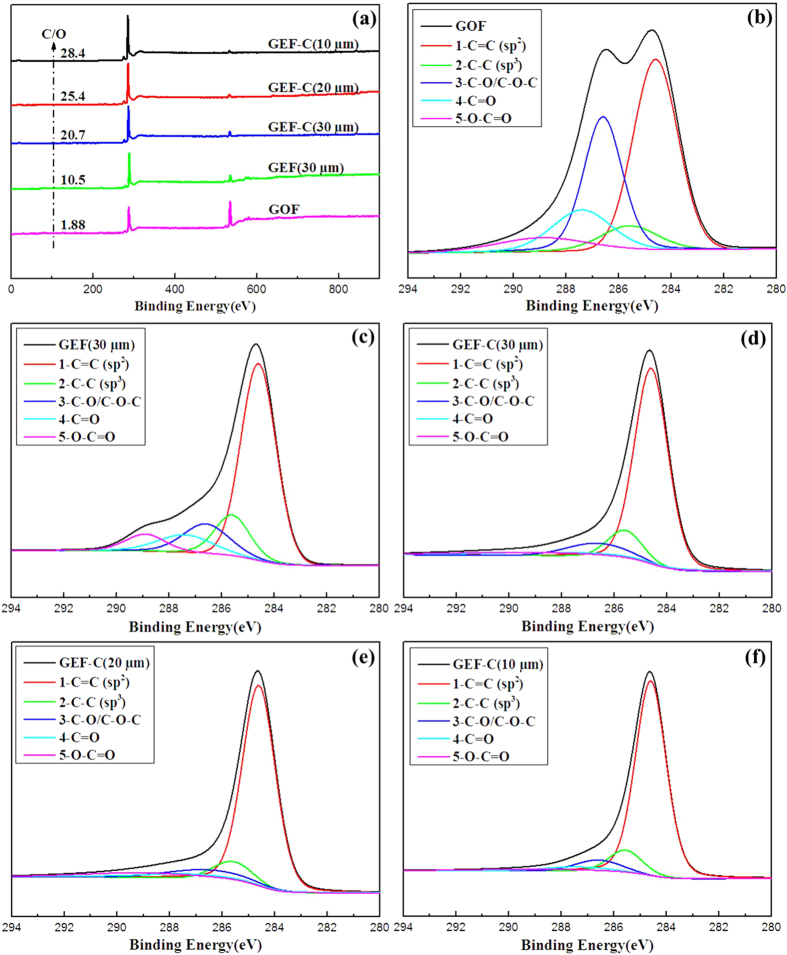
XP spectra analysis. (**a**) survey, C1s spectra of (**b**) GOF, (**c**) GEF(30 μm), (**d**) GEF-C(30 μm), (**e**) GEF-C(20 μm), (**f**) GEF-C(10 μm).

XPS analysis was carried out to obtain the evolution of functionalization in the film surface. By deconvolution of C1s region (Fig. 4), five different peaks that represent C=C (~284.6 eV, sp^2^), C–C (~285.6 eV, sp^3^), C–O (~286.1 eV, epoxy and hydroxyl), C=O (~287.4 eV, carbonyl) and O–C=O (~288.9 eV, carboxyl) were determined, which was similar to the previous reports^28^,^29^,^30^. As shown in Table 2, the relative C/O ratio was significantly increased from 1.9 for GOF to 10.5 for GEF, and 15.4, 20.7, 28.4 for GEF-C with thickness of 30 μm, 20 μm and 10 μm, respectively. It should be noted that the data from XPS was similar to that from elemental analysis for the C/O ratio of GOF, whereas the data from XPS was much larger than that from elemental analysis for the C/O ratio of GEF or GEF-C. It might be resulted from the more deep reduction on the surface of graphene oxide films in the H_2_-containing atmosphere at high temperature. In addition, the amounts of C=C components remarkably increased from 42.1 at.% for GOF to 61.8 at.% for GEF-30 μm and 70.3 at.% for GEF-C-30 μm, 75.0 at.% forGEF-C-20 μm, 76.7 at.% for GEF-C-10 μm, respectively. Meanwhile, the abundance of C–O, C=O and O–C=O related species correspondingly reduced. Correlated with the Raman spectra, XRD and TGA curves, the C1s peak fitting results indicated that the oxygen heteroatoms were removed from the basal plane of graphene oxide and C=C conjugated structure were restored during the thermal annealing reduction process in H_2_-containing atmosphere, especially with the Cu catalytic substrate for thinner.

**Figure 5:**
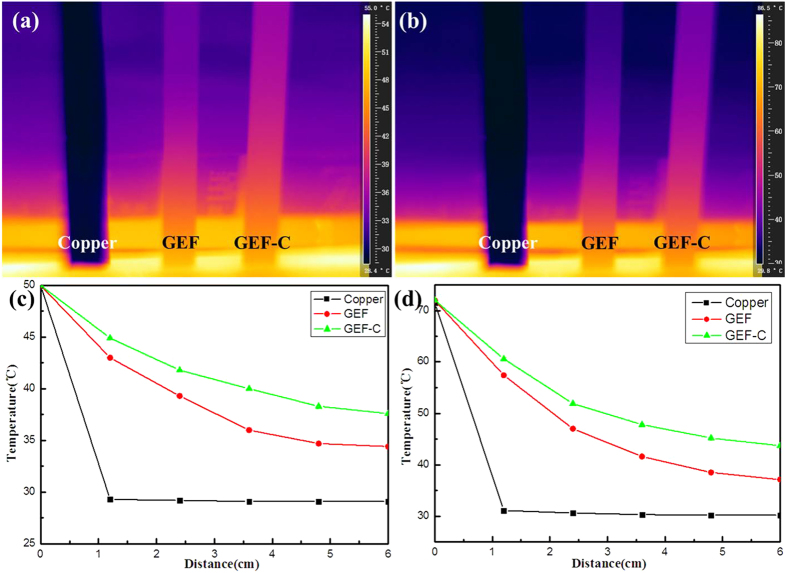
Images recorded by an infrared camera with samples attached to heating source. (**a,b**) Steady state temperature distribution of Cu foil, GEF, GEF-C around 50 °C and 72 °C. (**c,d)** The temperature profile at the length-line of the samples.

The thermal transport performances of Cu foil, GEF and GEF-C were compared by putting one side of the sample bars on the same temperature area of hot plate then measuring the temperature distribution along the bars with infrared camera. As shown in Fig. 5a,b, the heat transfer was much faster in GEF-C than GEF and Cu foil, suggesting the best thermal transport performance of GEF-C. The temperature profile at the length-line of the samples was calculated by FLIR quikerspot reporter. The temperature distribution of GEF-C has dropped from 50 °C on the hot source to 37.6 °C on the film bar end, or 72 °C on the hot source to 43.7 °C on the film bar end (Fig. 5c,d). However, the temperatures were reduced significantly from 50 °C to 34.4 °C, or 72 °C to 37.1 °C of GEF. In addition, the temperature of the Cu film bar was much lower than those of GEF-C and GEF. These results also implied that the GEF-C could transfer heat much fast than GEF and Cu.

**Figure 6:**
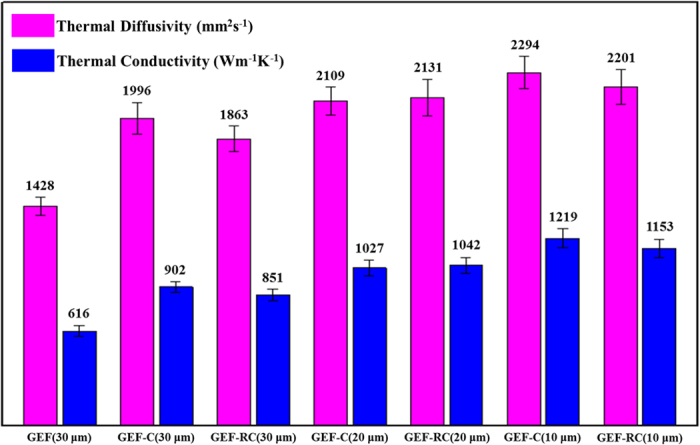
Thermal diffusivity and conductivity of GEF, GEF-C and GEF-RC.

The in-plane thermal diffusivity of films was measured using laser flash method. As summarized in Fig. 6, GEF-C with ~30 μm thickness displayed an outstanding thermal diffusivity and conductivity of 1996 mm^2^s^−1^ and 902 Wm^−1^K^−1^ at room temperature, which were considerably larger than those of ~30 μm thick GEF (1428 mm^2^s^−1^, 615 Wm^−1^K^−1^). Meanwhile, the electrical conductivity of the GEF-C was as high as ~3.6 × 10^4^ S/m relative to those of raw GOF (3.5 × 10^−3^ S/m) and GEF (2.6 × 10^4^ S/m). As discussed in the above, Cu foil could improve the reduction of graphene oxide films and the restoration of sp^2^ conjugated carbon lattice so as to enhance the electrical and thermal conductivity of final products. GEF-C also exhibited superior thermal conductivity of ~1027 Wm^−1^K^−1^ and ~1219 Wm^−1^K^−1^ with a smaller thickness of ~20 μm and ~10 μm, respectively. It might be mainly due to the following two points: first, the reduction degree of GEF-C could be increased with decreasing the film thickness; second, the catalytic effect of Cu would be more efficient for thinner layer of GOF since the function of catalysis on the interface. The GOF reduced on the second used Cu foil (GOF-RC) showed similar thermal conductivity to that of GOF reduced on the first used Cu foil, confirming the function of recyclable Cu foils in the enhanced reduction of graphene oxide films at high temperature in the H_2_-containing atmosphere.

**Figure 7:**
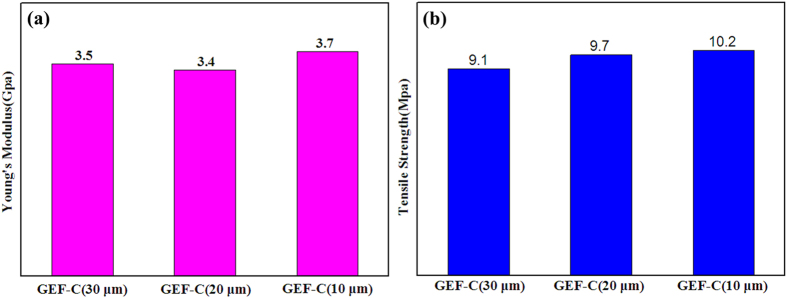
Mechanical properties of GEF-C at different thickness. (**a**) Young’s Modulus, and (**b**) Tensile Strength.

Although the thermal conductivity of the as-prepared graphene films was lower than that of the suspended individual monolayer graphene (~5300 Wm^−1^K^−1^) [11], the practical applications need macroscopic materials such as films assembled by a large number of graphene nanosheets for large-volume heat dissipation^9^. Furthermore, the as-prepared graphene films in this work demonstrated outstanding thermal conductive performance relative to the reported graphene based films (papers). For example, Ar^+^ ion irradiating reduced graphene oxide films showed 0.198 Wm^−1^K^−1^ although with high C/O ratio and electrical conductivity^31^. Graphene films assembled by a vacuum filtration method showed thermal conductivity of 112 Wm^−1^K^−1 ^9. The graphene-graphene composite films reduced from graphene oxide at 1060 °C showed only 220 Wm^−1^K^−1 ^32. The 1030 °C CVD graphene-Cu-graphene composite films exhibited ~370 Wm^−1^K^−1 ^33. These thermal conductivities were much lower than that of the GEF-C films in this work (~1219 Wm^−1^K^−1^). The graphene oxide films reduced at 2000 °C possessed high thermal conductivity of ~1100 Wm^−1^K^−1^ with 8.4 μm thickness^4^. The reduced graphene oxide films could show excellent thermal conductivity of ~1434 Wm^−1^K^−1^, but it needed graphitized at very high temperature of 2850 °C^3^. In this work, the graphene oxide films reduced at much low temperature of 900 °C on Cu catalytic substrate demonstrated comparable high thermal conductivity to these graphene films reduced at very high temperature more than 2000 °C. The as-prepared graphene films also demonstrated extremely higher thermal conductivity than the one of the best metallic conductive conductor of Cu (~400 Wm^−1^K^−1^)^1^. In addition, the as-prepared graphene films possessed greatly lighter weight with density of 0.8 g/cm^3^ than that of Cu (8.9 g/cm^3^), which was beneficial to its application in portable devices. Due to the cheap graphite raw materials and low temperature process, the reduced graphene oxide films on recyclable Cu substrate for thermal conductive materials were also more cost effective in comparison with the current commercial graphitic papers typically prepared by carbonization process from expensive polyimide films at very high temperature of usually about 3000 °C, or the thermal conductive papers fabricated by CVD graphene nanosheets. The mechanical properties of GEF-C at different thickness were investigated by a tensile test. As shown in Fig. 7, the Young’s modulus and tensile strength were evaluated to be ~10 GPa and ~3.5 MPa, exhibiting acceptable mechanical performance for practical application. The good mechanical properties of GEF-C might be attributed to the restoration of graphene sheets with elimination of oxygen groups, decreased interlayer spacing and enhanced interlayer contact during thermal annealing with assistance of Cu foil.

## Discussion

**Figure 8:**
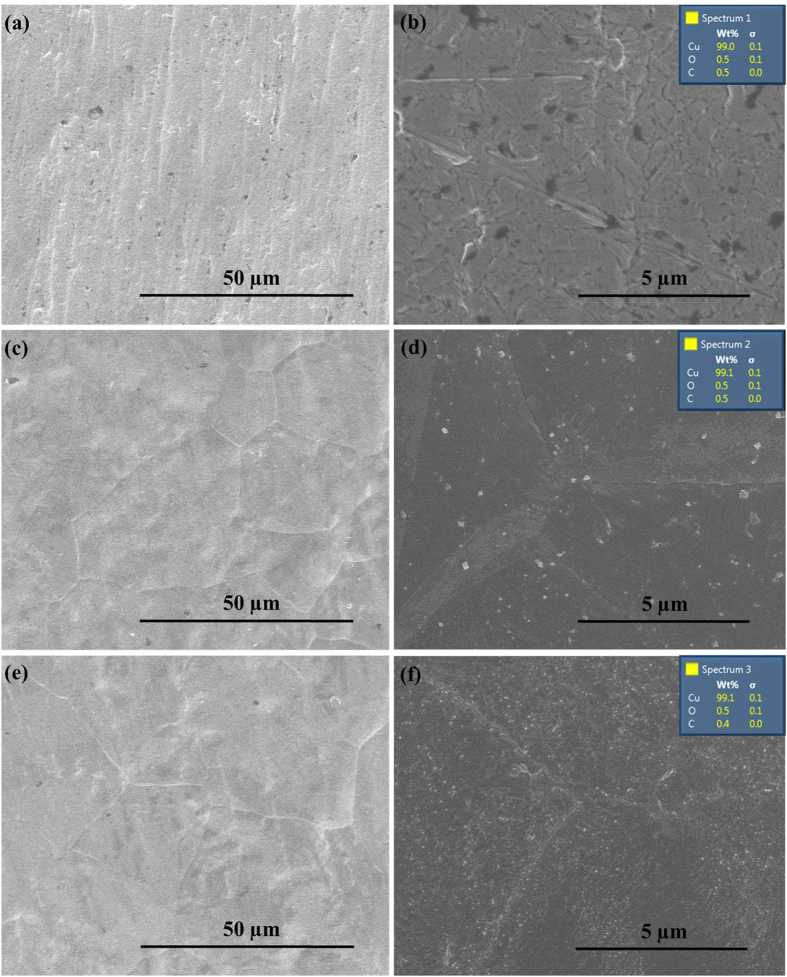
SEM images of different Cu foils. (**a**,**b**) SEM images of Cu foil, (**c**,**d**) SEM images of Cu foil after annealing, (**e**,**f**) SEM images of Cu/graphene foil after annealing, (inset) EDX results of the samples.

**Figure 9:**
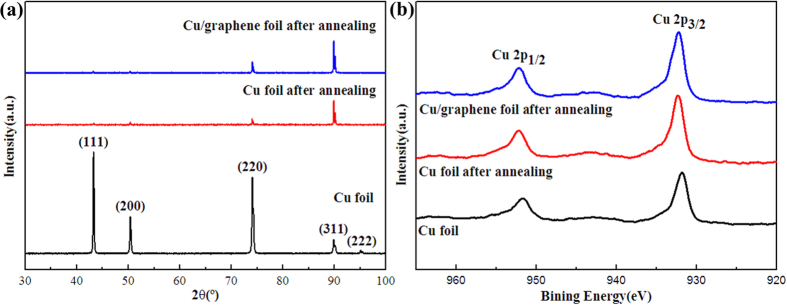
Structure evolution of different Cu foil. (**a**) XRD patterns and (**b**) XP spectra of Cu2p of Cu foil, Cu foil after annealing, Cu/graphene foil after annealing.

**Figure 10:**
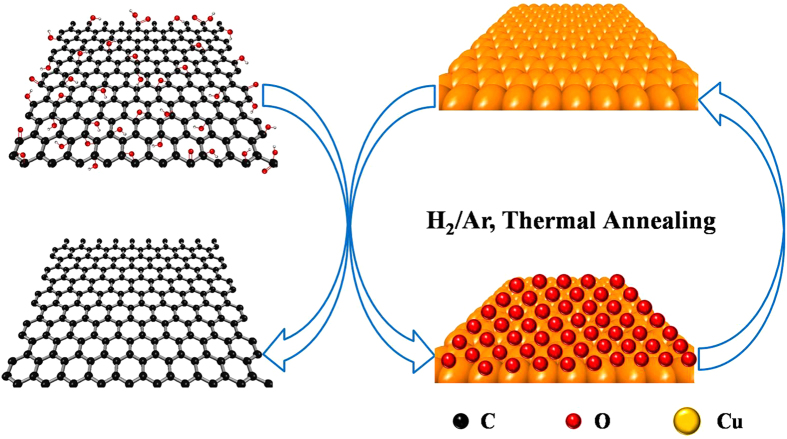
Illustration of the preparation of graphene based on Cu foil reduction.

To further investigate the effect of Cu foil in the reduction of graphene oxide films, the microscopic morphology and structure evolution of original Cu foil, bare Cu foil and Cu foil substrate for GO reduction after annealing at 900 °C in H_2_-containing atmosphere were characterized. Cu foil substrate for GO reduction was also named as Cu/graphene foil in Figs 8, 9, 10 since it was obtained by peeling off graphene film after annealing at 900 °C in H_2_-containing atmosphere. As shown in Fig. 8, bare Cu foil and Cu foil for GO reduction illustrated similar surface morphology with broader and larger size grains after annealing relative to the original Cu foil. This is mainly attributed to the recrystallization of Cu grain at high temperature in H_2_-containing atmosphere. The element composition of the samples can be observed from the EDX results as shown in the insets of Fig. 8. Cu (about 99%) was the major element with less existence of C and O elements in all Cu foils. Figure 9 shows XRD and XPS results of the original Cu foil, bare Cu foil and Cu foil for GO reduction after annealing at 900 °C in H_2_-containing atmosphere. Bare Cu foil and Cu foil for GO reduction after annealing display the same crystalline phase whereas different from that of the original Cu foil (Fig. 9a). The reason is that the thermal annealing can bring a preferential orientation of metallic Cu like the previous studies^34^. The Cu_2p_ XPS spectra (Fig. 9b) for the three samples were almost same and the main peaks were observed at BE (2p_1/2_) = 952.2 eV and BE (2p_3/2_) = 932.3 eV, with shake-up satellites. All the above SEM/XRD/XPS results show that there was no difference between bare Cu foil and Cu foil for GO reduction after annealing at 900 °C in H_2_-containing atmosphere, suggesting the Cu as a catalyst in the GO reduction. A possible mechanism was proposed in Fig. 10 for the catalytic reduction of GO on Cu foil at high temperature in H_2_-containing atmosphere. Graphene oxide could be reduced by metallic Cu foil to form graphene film and Cu oxide due to the different potential^35^,^36^,^37^. H_2_ could also reduce graphene oxide to graphene at high temperature^38^. Therefore, graphene oxide can be efficiently reduced to graphene with the synergistic reduction effect of Cu and H_2_ at high temperature. On the other hand, Cu oxide could be easily reduced to metallic Cu at high temperature in the H_2_-containing atmosphere then for next reduction of GO. It means that Cu could be used as recyclable catalysts for reduction of GO films.

## Conclusions

A facile and cost-effective approach was developed to fabricate large area free standing graphene films with high C/O ratio from graphene oxide films using recyclable Cu foil as catalytic substrate at high temperature in H_2_-containing atmosphere. Comparing to the graphene oxide films reduction without Cu foil, the graphene oxide films reduction on Cu foil were very effective to improve the thermal/electrical conductivity upon the recovery of defects and removal of oxygen-containing functional groups in the highly aligned graphene films. Furthermore, the graphene films reduction on Cu foils demonstrated deeper reduction degree and higher thermal conductivity with the thinner thickness. In addition, the graphene films displayed favorable mechanical property and excellent flexibility.

## Methods

### Graphene film fabrication

Graphite oxide was synthesized from natural graphite by using a modified Hummers method^39^. Then the colloidal dispersion of graphene oxide was obtained by ultrasonic exfoliation (Kunshan KQ3200DB, 1000 W) of graphite oxide in deionized water, followed by mild centrifugation (2500 rpm, 10 minutes) to remove non-exfoliated sheets. Graphene oxide films were obtained by injecting Cu foils into GO suspension and kept in evaporation of water under appropriate heating (50 °C) for films formation until drying. The obtained composite films or bare GO films were hot pressed between two aluminum oxide plates at 150 °C for 1 h, followed by thermal treatment in 5%H_2_-Ar gas mixture as following: the films were heated from room temperature to 300 °C at the rate of 5 °C/min, and kept at 300 °C for 30 min; then quickly to 900 °C (10 °C/min), followed by thermal annealing at 900 °C for 1 h; last, the films were naturally cooled down to room temperature. After easily peeled off from the Cu substrates, free-standing graphene films were obtained. The graphene films obtained with and without Cu substrate are abbreviated GEF-C and GEF, respectively.

### Sample characterization

The morphologic micrographs of the obtained samples were characterized by scanning electron microscope operated at 5.0 kV (SEM, FEI Nova NanoSEM 450). Raman spectra were recorded by Renishaw in Via Raman microscope system with 514-nm laser excitation. X-ray diffraction (XRD) measurements were carried out by using a D/Max-3c X-ray diffractometer with Cu K (1.5406 Å), with an operation voltage and current of 40 kV and 40 mA, respectively. The as-obtained samples were characterized using thermal gravimetric analysis (TGA) system (SDT Q600, TA, USA). The carbon and oxygen content of the films was conducted by vario EL elemental analyzer (Elemental Analysensysteme GmbH, Germany). X-ray photoelectron Spectroscopy (XPS) was obtained by AXIS ULTRA DLD (Ktatos, England). Thermal diffusivity (α) of the film was measured by laser flash thermal analyzer (LFA447/2-2lnsb NanoFlash). The specific heat (*C*_*p*_) used in this paper was measured by differential scanning calorimeter (DSC, 200 F3). The density (ρ) was calculated according to the formulas ρ = *m*/*V*. The 25.4 mm diameter disk film was punched out for the in-plane thermal diffusivity measurement. The thickness (*h*) of these films was measured with SEM. The volume (*V*) was obtained according to the formulas *V* = π×12.7×12.7×*h* = 506.45×*h*. The thermal conductivity (*K*) was calculated from *K* = α·*C*_*p*_·ρ. A FLIR T335 infrared camera was further used to record the thermal transport performances of Cu foil, GEF and GEF-C, and temperature profile at the length-line of the samples were calculated by the FLIR quickplot reporter. In the experiments, both top and bottom surfaces attached the heat source were coated with a thin layer thermal grease to reduce interface resistance and ensure the same temperature for the samples. The mechanical properties were investigated by dynamic mechanical analyzer (DMA Q800, TA instruments).

## Additional Information

**How to cite this article**: Huang, S.-Y. *et al.* Enhanced Reduction of Graphene Oxide on Recyclable Cu Foils to Fabricate Graphene Films with Superior Thermal Conductivity. *Sci. Rep.* **5**, 14260; doi: 10.1038/srep14260 (2015).

## Supplementary Material

Supplementary Information

## Figures and Tables

**Table 1:** Summary of elemental composition of the films.

**Sample**	**C**^a^ **(at%)**	**O**^a^ **(at%)**	**C/O**^a^ **ratio**	**d**^b^ **(Å)**	**Residual carbon**^c^ **(%)**
GOF	64.3	35.7	1.8	7.5	34.8
GEF(30 μm)	91.0	9.0	10.1	3.5	86.4
GEF-C(30 μm)	93.5	6.5	14.4	3.4	91.5
GEF-C(20 μm)	95.2	4.8	19.8	3.4	93.2
GEF-C(10 μm)	95.9	4.1	23.4	3.4	95.2

^a^Determined by element analysis.

^b^Determined by XRD.

^c^Determined by TGA.

**Table 2:** Fitted results of C1s XPS spectra of GOF, GEF and GEF-C.

**B.E.(eV)**	**C1(284.6)**	**C2(285.6)**	**C3(286.6)**	**C4(287.4)**	**C5(288.9)**	
Assignment	C=C(sp^2^)	C–C(sp^3^)	C–O	C=O	C(O)O	C/O
GOF	42.1	9.2	28.2	13.4	7.1	1.9
GEF(30 μm)	61.8	13.1	12.7	7.0	5.4	10.5
GEF-C(30 μm)	70.3	10.6	8.5	5.7	4.9	15.4
GEF-C(20 μm)	75.0	8.1	6.9	5.2	4.8	20.7
GEF-C(10 μm)	76.7	8.0	6.5	4.3	4.5	28.4
