# Supplementary material for: Enhanced Reduction of Graphene Oxide on Recyclable Cu Foils to Fabricate Graphene Films with Superior Thermal Conductivity
Source: Sci Rep. 2015 Sep 25;5:14260. doi: 10.1038/srep14260 (PMC4585894; doi:10.1038/srep14260)
Supplement: Supplementary Information [file srep14260-s1.pdf]

Enhanced Reduction of Graphene Oxide on Recyclable Cu Foils to Fabricate Graphene Films with Superior Thermal Conductivity

Sheng-Yun Huang[1,2], Bo Zhao[1,2], Kai Zhang[3], Matthew M.F. Yuen[3],Jian-Bin Xu[4], Xian-Zhu Fu[1,2,*], Rong Sun[1,2,*], and Ching-Ping Wong[4,5]

1 Shenzhen Institutes of Advanced Technology, Chinese Academy of Sciences, Shenzhen, 518055, P. R. China E-mail: xz.fu@siat.ac.cn and rong.sun@siat.ac.cn

[2] Shenzhen High Density Electronic Packaging and Device Assembly Key Laboratory Shenzhen, 518055, China

[3] Department of Mechanical Engineering, Hong Kong University of Science and Technology, Hong Kong, China

[4] Department of Electronics Engineering, The Chinese University of Hong Kong, Hong Kong, China

[5] School of Materials Science and Engineering, Georgia Institute of Technology, Atlanta, Georgia 30332-0245, United States

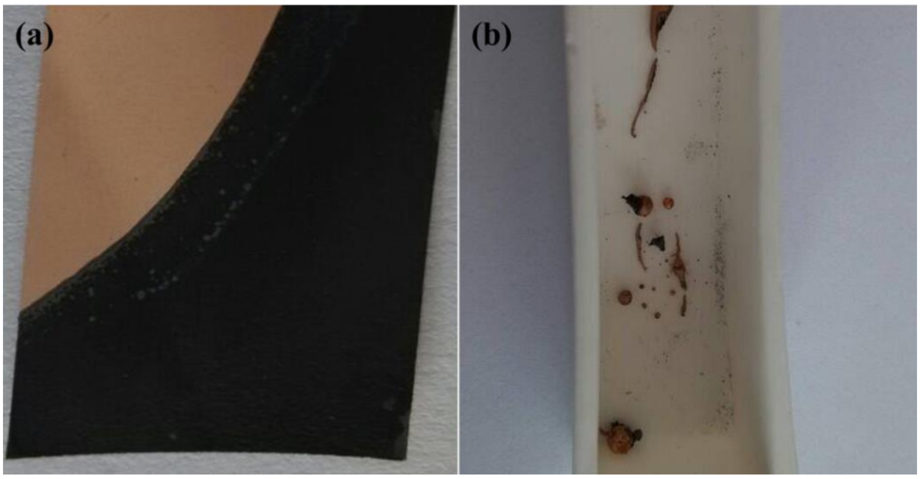

Figure 1S | Digital photographs of GOF on Cu foil (a) before and (b) after annealing at 1000 ^{o}C.
